# On Measuring Implant Fixation Stability in ACL Reconstruction

**DOI:** 10.3390/s21196632

**Published:** 2021-10-06

**Authors:** Emir Benca, Ivan Zderic, Jan Caspar, Kenneth van Knegsel, Lena Hirtler, Boyko Gueorguiev, Harald Widhalm, Reinhard Windhager, Peter Varga

**Affiliations:** 1 Department of Orthopedics and Trauma Surgery, Medical University of Vienna, 1090 Vienna, Austria; harald.widhalm@meduniwien.ac.at (H.W.); reinhard.windhager@meduniwien.ac.at (R.W.); 2 AO Research Institute Davos, 7270 Davos, Switzerland; ivan.zderic@aofoundation.org (I.Z.); jan.caspar@aofoundation.org (J.C.); kenneth.vanknegsel@luks.ch (K.v.K.); boyko.gueorguiev@aofoundation.org (B.G.); peter.varga@aofoundation.org (P.V.); 3 Department of Orthopedics and Trauma Surgery, Cantonal Hospital of Lucerne, 6000 Lucerne, Switzerland; 4 Center for Anatomy and Cell Biology, Division of Anatomy, Medical University of Vienna, 1090 Vienna, Austria; lena.hirtler@meduniwien.ac.at

**Keywords:** ACL, stiffness, slippage, strain, interference screw, biomechanical testing, motion tracking, human specimens, in vitro study

## Abstract

Numerous methods and devices are available for implant fixation in anterior cruciate ligament (ACL) reconstruction. Biomechanical data indicate high variability in fixation stability across different devices. This study aims to provide a better insight into measuring the structural characteristics and mechanical behavior of ACL implant fixations. Fourteen human tibial specimens with reconstructed ACLs were subjected to progressively increasing dynamic loading until failure. The motions of the tibia, the proximal and distal graft ends, as well as the testing frame and actuator, were continuously recorded via a motion tracking system. Significantly higher displacements of the machine actuator (1.0 mm at graft slippage onset, and 12.2 mm at ultimate load) were measured compared to the displacements of the proximal (0.8 and 4.3 mm, respectively) and distal graft (0.1 and 3.4 mm, respectively) ends. The displacements measured at different sites showed significant correlations. The provided data suggest significant and systematic inaccuracies in the stiffness and slippage of the fixation when using machine displacement, as commonly reported in the literature. The assessment of the distal graft displacement excludes the artifactual graft elongation, and most accurately reflects the graft slippage onset indicating clinical failure. Considering the high displacement at the ultimate load, the ultimate load could be used as a standardized variable to compare different fixation methods. However, the ultimate load alone is not sufficient to qualitatively describe fixation stability.

## 1. Introduction

The rupture of the anterior cruciate ligament (ACL) is among the most common knee injuries. Complete ruptures are typically treated surgically [1,2]. An ACL rupture substantially increases the anterior tibial translation and the internal rotation and, consequently, shifts the stress and strain areas in the knee articular cartilage [3,4]. The altered kinematics in the knee joint following an ACL rupture are likely the cause for the increased risk of osteoarthritis in the cartilage, especially in the medial compartment [5]. An ACL reconstruction involves: the removal of the damaged ACL; preparation of the harvested auto- or allograft; drilling of the tibial and femoral tunnels; placement of the graft in an anatomically similar, or different, position to the original ACL; and the fixation of the graft. Numerous methods and devices for ACL graft fixation are currently available, including mulch screws, buttons, cross pin systems, interference screws, metal screws, staples, and washers [6]. However, there is no clinical consensus regarding the optimal fixation [7], nor with regard to mechanical stability. Some previous studies have evaluated the stability following different fixation methods using typically animal bone and human graft specimens [8,9,10,11,12,13,14,15,16]. The assessed structural characteristics of the fixations included slippage onset, stiffness, yield, and ultimate load. These measures can be used for direct performance comparisons between different devices and methods for ACL graft fixation. In short, slippage (in mm),the relative movement of the tendon graft to the bone, is also used clinically to define the onset of graft loosening. A low cyclic slippage is a necessary feature of any fixation device or system used to secure a reconstructed ACL [7]. Stiffness (in N/mm) describes the magnitude of bone-fixation and device-tendon complex resistance against deformation in response to the applied loads in the elastic range, i.e., in a reversible loading regime. Stiffness of the fixation is a clinically important parameter reflecting the stability of the treated knee joint. Yield load (in N) marks the limit of the elastic behavior and the onset of irreversible plastic deformation. Usually, the latter results from either slippage or the mechanical damage of the graft. Finally, the ultimate load (in N), being the absolute maximum of the load-displacement curve, defines the load level at which the catastrophic construct failure occurs. Clinically, this would result in a traumatic injury rather than a failure that accumulated gradually during the rehabilitation phase, with partial weight-bearing and limited range of motion. The aforementioned mechanical characteristics can, in most cases, be derived from the load-displacement curves of the machine transducers, recorded by the controllers during destructive tensile testing. However, the transducer displacement does not correspond to the displacement of the graft, but superimposes the displacements and compliances of all of the components of the experimental setup, including loading frame, embedding, bone, implant fixation, and graft, as well as the interfaces between these components. Therefore, evaluating transducer displacement to evaluate the stability of different methods to fix the ACL graft in the bone tunnel, as commonly performed in biomechanical studies, might be the potential source of an error of a yet unknown magnitude. To the best knowledge of the authors, the relation of machine displacement to the actual displacement of the tendon graft has not been addressed in any previously published study. Motion tracking methods enable the assessment of the relative displacements and movements between the components, independently of the testing frame movements. This allows for control over the confounding factors of the test setup, and enables the testing of the unbiased effect of the isolated fixation construct. Therefore, this study aimed to: (1) measure the isolated displacements and elasticities of single components in the experimental setup via motion tracking; (2) compare the magnitude of the measured machine displacement and the actual slippage of the ACL graft; and finally, (3) investigate if stiffness and ultimate load can be valid parameters to quantify the primary stability of an ACL fixation.

## 2. Materials and Methods

### 2.1. Specimen Preparation

Fourteen anatomic specimens of human tibiae, with preserved semitendinosus and gracilis tendons, were obtained from 3 female and 8 male donors (age 72.5 ± 5.7 years (mean ± standard deviation), range 62–79 years). The fresh-frozen specimens were thawed at room temperature for 24 h prior to preparation. They were also kept moist with a 0.9% saline solution between the preparation steps, and tested at room temperature. The most proximal part of each tibia was cut at a total length of 110 mm using an oscillating saw, and carefully stripped of soft tissue. Following the surgical procedure (2.2), the distal 30 mm was embedded in polymethyl methacrylate (PMMA, SCS-Beracryl D28, Suter Kunststoffe AG, Fraubrunnen, Switzerland) cylinders of 80 mm diameter and 28 mm height. The embedding was secured in the cup by means of twelve 5 mm screws with a conical tip. Projected line laser beams ensured that the graft tunnel and the graft were aligned with the vertical axis representing the biomechanical worst-case scenario.

### 2.2. Surgical Procedure

The semitendinosus and gracilis tendons were utilized in all specimens as ACL replacements. The tendon grafts were harvested in their full length, quadrupled and, if necessary, longitudinally trimmed to match the 8 mm diameter sizing sleeve. The distal 30 to 40 mm of the tendon grafts were then sutured in a whipstitch manner using No. 1 Polysorb suture (Covidien Ilc, Mansfield, MA, USA), and pretensioned at 50 N. A guide wire was placed anatomically in the proximal tibia in lateral to the medial direction at an angle of 55°, followed by the incremental drilling of the bone tunnel diameter from 5 mm up to 8 mm. The graft was inserted into the distal end of the tunnel and pulled in proximal direction. A 1.0 mm guide wire was placed laterally at the interface between the tendon graft and the tunnel followed by the placement of a fully threaded 8 × 28 mm BioComposite Interference Screw (Arthrex Inc., Naples, FL, USA), or an 8.0 mm Shark Screw ACL (surgebright GmbH, Lichtenberg bei Linz, Austria), designed for ACL fixation over the same guide wire. The presented samples are a subpopulation from a study evaluating the fixation stability of different fixation devices (data to be published).

### 2.3. Biomechanical Testing

**Figure 1:**
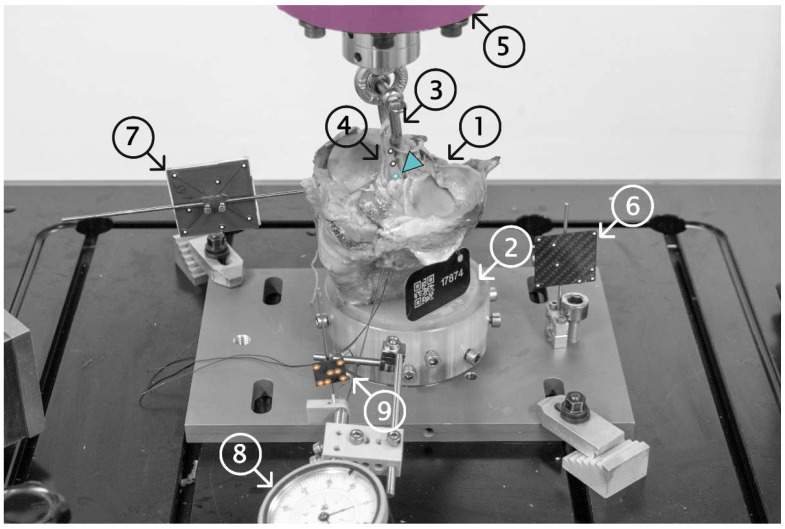
The proximal 110 mm of the anatomic human tibia specimens (1) were distally potted using PMMA (2) and fixed into the load frame. A 5.5 mm D-shackle (3) was inserted and secured into the loop of the tendon graft (4) and attached to the machine actuator (5). The components of interest were marked using self-adhesive point markers (load frame (6), tibia specimens (7), and tendon grafts (4)) and their spatial motions were tracked using a stereographic optical motion tracking system. A dial gauge (8), rigidly fixed to the load frame, was used to pretension the suture at the distal end of the tendon graft (9), and was tracked to monitor the migration of the tendon fixation screw in the tunnel (slippage), which was not visible to the tracking system. Colors indicate the site for assessed displacements (from proximal to distal: machine (pink); proximal- (cyan); and distal (orange) tendon grafts).

As visualized in Figure 1 the specimens (part (1)) embedded distally in PMMA (2) were mounted in an electrodynamic test system (Acumen; MTS Systems Corporation, Eden Prairie, MN, USA). A D-shackle (3) was looped through the quadrupled tendon graft (4) and attached to the machine actuator (5). In biomechanical studies, typically, the specimen and the fixation are exposed to cyclic tensile loading, e.g., 1000 cycles of force-controlled dynamic loading with increasing amplitude from 50 to 250 N, followed by quasi-static load-to-failure testing [17,18,19,20,21]. The reported screw fixation stiffnesses ranged between 80 and 162 N/mm, and the ultimate loads ranged between 285 and 864 N [8,9,10,11,12,13,21,22,23]. Following this methodology, the tensile load was applied, in the present study, parallel to the graft tunnel, mimicking the worst-case scenario. In accordance with similar studies, the specimens were preloaded at 50 N to eliminate any potential settling effects and, afterwards, loaded cyclically at a rate of 1 Hz, with a sinusoidal profile at a constant valley load of 50 N, and a peak load level starting from 50 N, and increasing at a rate of 0.1 N/cycle. The test was stopped when catastrophic construct failure occurred. No isolated ultimate load test, mimicking a traumatic event, was performed in order to achieve construct failure during cyclic loading in all specimens. ACL re-ruptures are reported to occur 3.5 [24] to 53 [25] months after surgery, and repetitive loading seems to more accurately reflect the clinical reality in primary stability testing. The load and actuator displacement were digitalized with a sample rate of 128 Hz. The relative motion of the graft at the proximal tibial tunnel to the bone ((4) to (7)), bone to the load frame ((7) to (6)), and suture connected via a dial gauge (8) rigidly fixed to the load frame, to the distal graft end of the tibial tunnel to the bone ((9) to (7)), were measured via stereographic optical motion tracking (Aramis SRX; GOM GmbH, Braunschweig, Germany) at a rate of up to 115 Hz. The system’s two 12 megapixel optical cameras continuously recorded the positions of the markers attached to each component. The tracking of the suture at the distal end of the graft enabled the direct monitoring of graft slippage with respect to the host bone. Slippage onset was defined when this relative displacement exceeded 0.1 mm at peak load. The latter threshold was chosen as the lowest detectable displacement limit that could clearly be distinguished from signal noise. Clinically, this value represents the onset of clinical failure.

### 2.4. Statistical Analysis

For data analysis, displacements at two events, namely, at the onset of slippage and when reaching the ultimate load, were assessed from continuously recorded data. The statistical analysis was performed using the SPSS software package (v. 27, IBM SPSS Corp., Armonk, NY, USA). The data of each outcome measure were tested for normality of distribution using the Shapiro-Wilk test. Given the non-normal distribution (NND) for most variables, the Wilcoxon signed rank test was performed to test for statistically significant differences between the outcomes measured at the different locations of the test setup. Monotonic regression analysis was performed, and Spearman’s correlation coefficients (r_S_) were computed to investigate the strength of correlation between the displacement measured at the different test setup locations, at slippage onset and ultimate load. Machine and proximal graft end displacement, as well as graft strain, were considered dependent variables. The significance level was set to *p* = 0.05 for all statistical tests. Data obtained from the specimens with two different implant fixations were pooled. Values described in the text are mean and standard deviations (SD).

## 3. Results

The median machine displacement at ultimate load reached 12 mm. The median displacement of the proximal graft end was 4 mm, and that of the distal end was 3 mm at the ultimate load (see Table 1). The median absolute vertical displacement of the bone-specimen-embedding complex was less than 1 mm and showed low deviation. Thus, it did not significantly affect the overall displacement. The graft strains showed high deviations with a maximum of 14.5% at the ultimate load. 

**Figure 2:**
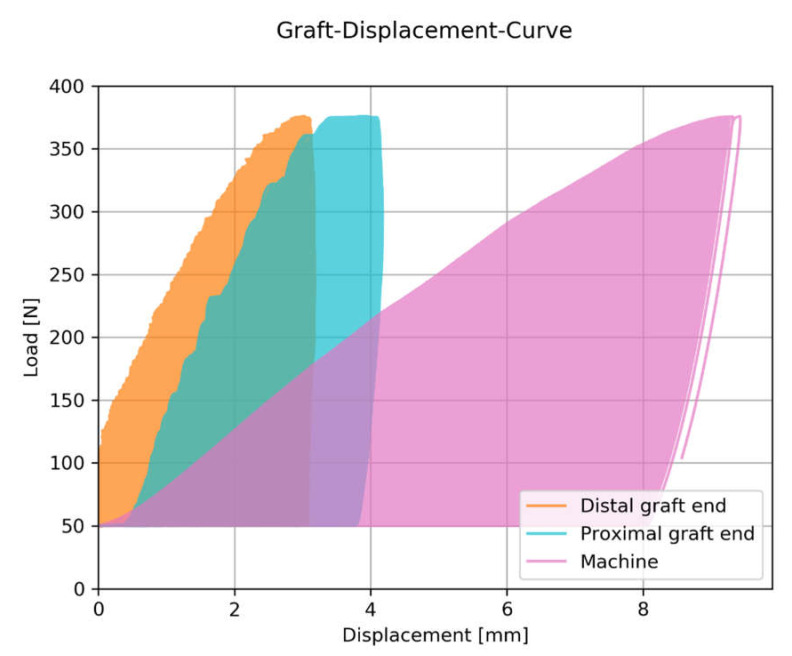
Typical load-displacement curves of a sample. The displacements at the distal and proximal end of the tendon graft were measured by an optical motion tracking system. The machine displacement was assessed by the integrated displacement transducer.

A typical load-displacement curve of a single sample is displayed in Figure 2. While the machine displacement linearly increases to approximately 9.4 mm, or 376 N ultimate load (maximum value in the graph), the displacements of the graft ends are significantly lower. The onset of slippage can be identified in the onset of displacement in the curve of the distal graft end at 70 N of tensile load.

**Figure 3:**
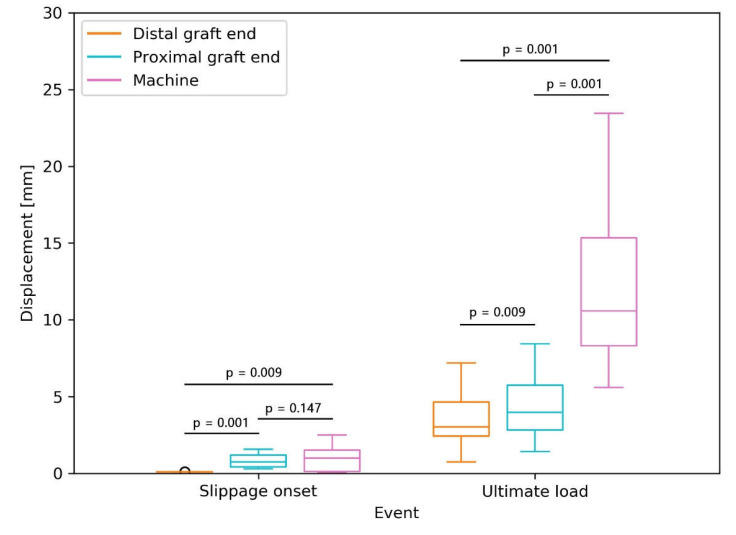
Boxplots for displacements of the proximal and distal graft ends, and the machine actuator assessed at the events of slippage onset and reaching the ultimate load. Each box denotes the 25th through 75th percentiles, the horizontal bar within the box denotes the median value, and the whiskers extend 1.5 times the interquartile range above and below the 75th and 25th percentiles, respectively. Observations beyond the whiskers are plotted individually.

At the slippage onset, there was no significant difference between the displacements of the proximal graft end and of the machine actuator (*p* = 0.147, Figure 3). The displacement of the distal graft end was significantly lower than the displacements of the proximal graft end and of the machine actuator (*p* = 0.001 and *p* = 0.009, respectively). The displacement at ultimate load, measured by the machine, was significantly higher than the displacements of both the proximal and distal graft ends (both *p* = 0.001). The proximal graft end displacement was significantly higher than the distal one (*p* = 0.009).

**Figure 4:**
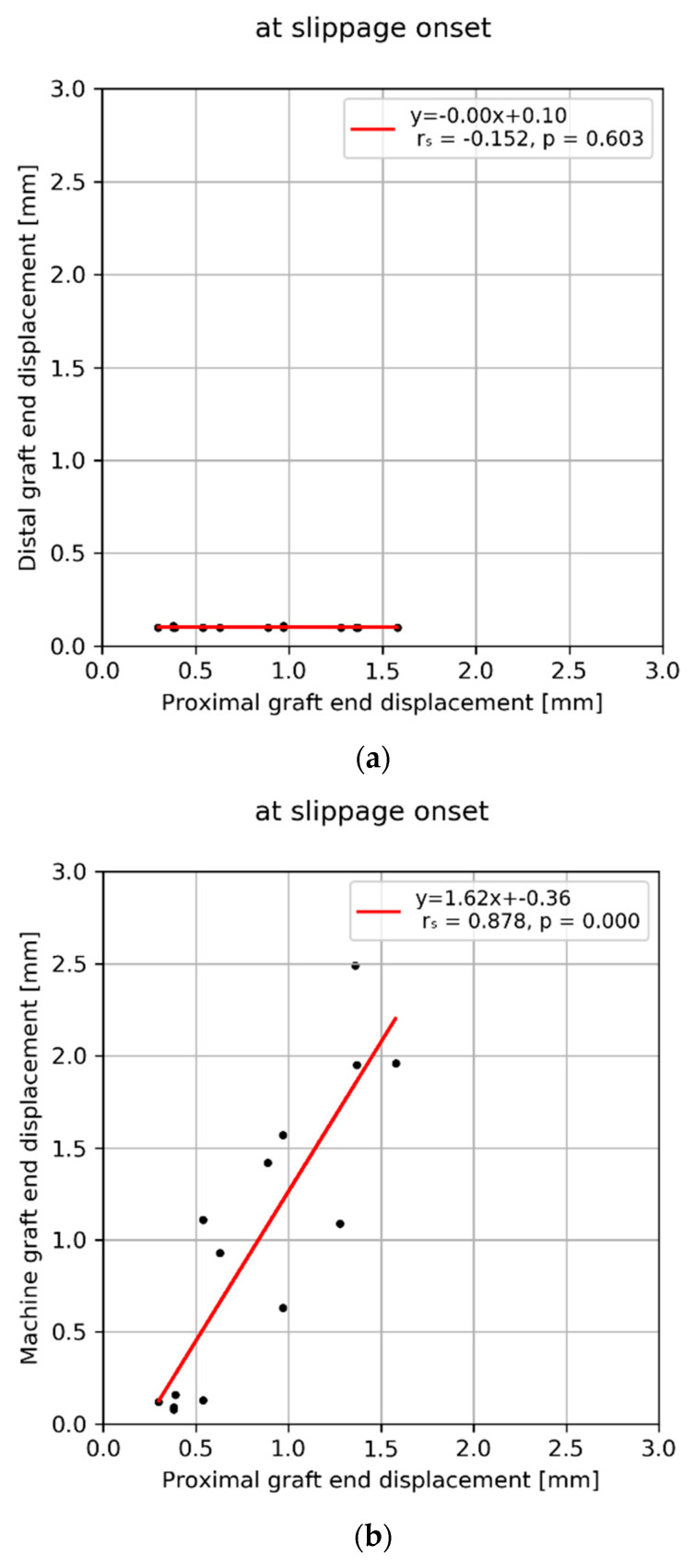

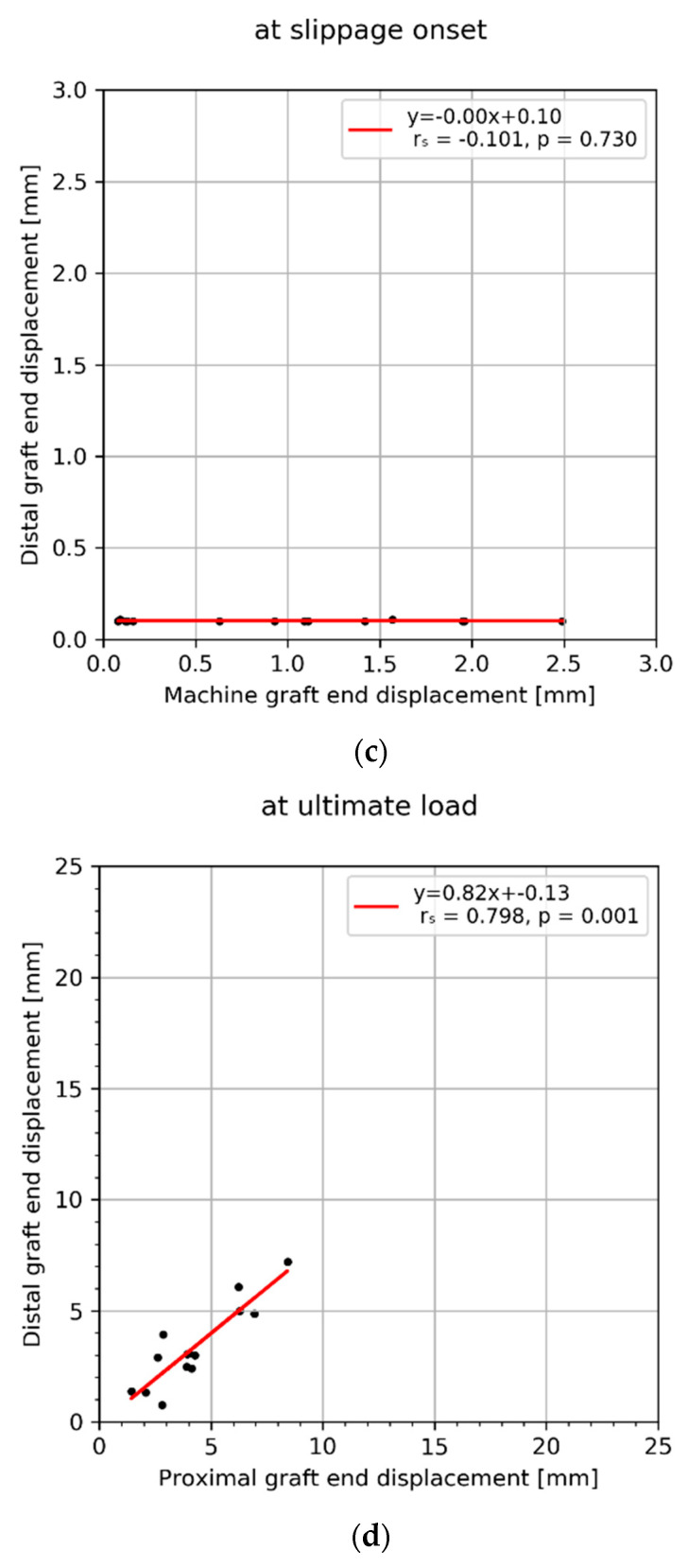

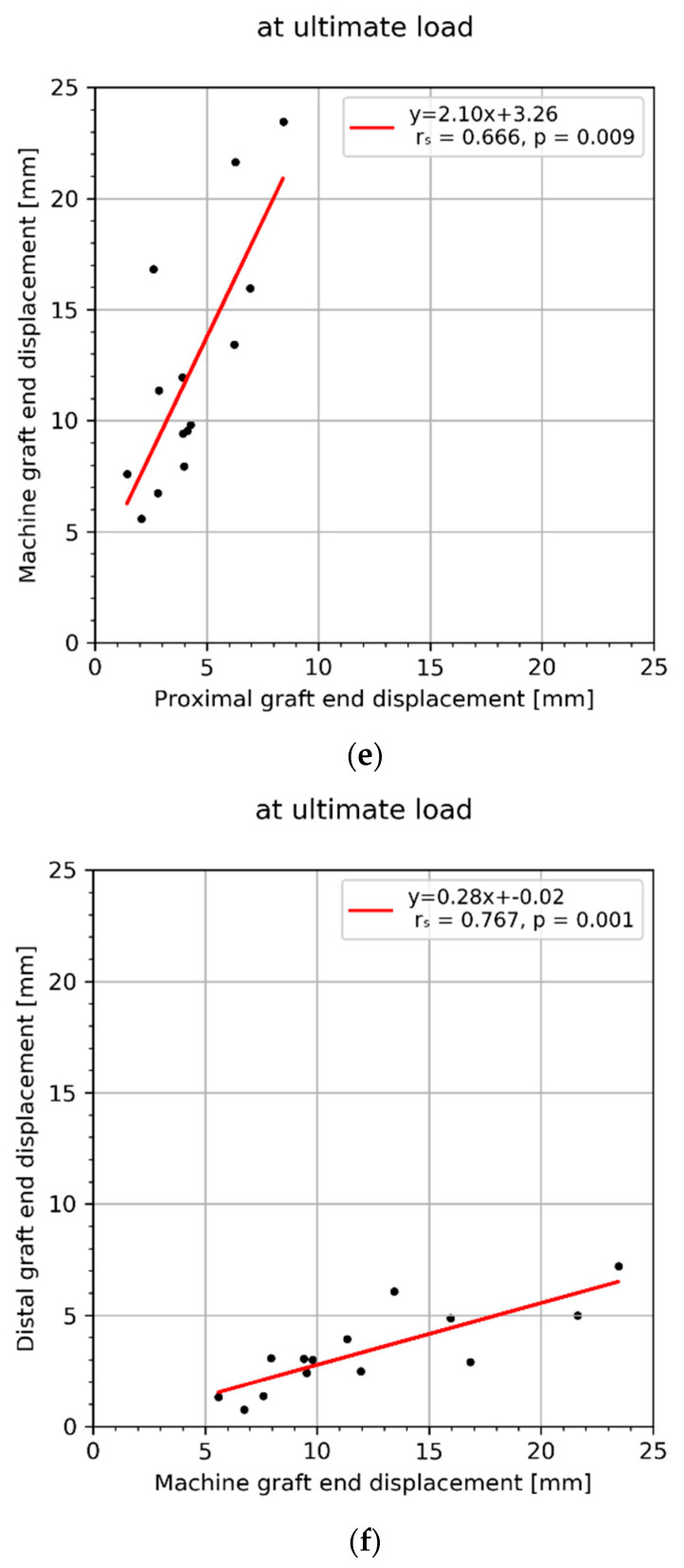
Correlation plots between displacements of the proximal and distal graft ends and the machine transducer assessed at slippage onset (**a**–**c**) and ultimate load (**d**–**f**).

Slippage onset was defined as a displacement of the distal graft end >0.1 mm. Hence, the value for the distal graft end displacement remained constant across all specimens. At slippage onset, no correlation was observed between either the proximal graft end (Figure 4a), or the machine actuator displacement (Figure 4c) and distal graft end. All other displacement combinations at slippage onset, proximal graft end vs. machine (Figure 4b), as well as at ultimate load, proximal vs. distal graft ends (Figure 4d), proximal vs. machine (Figure 4e), and distal vs. machine (Figure 4f) showed significant correlations (−0.152 < r < 0.878).

**Figure 5:**
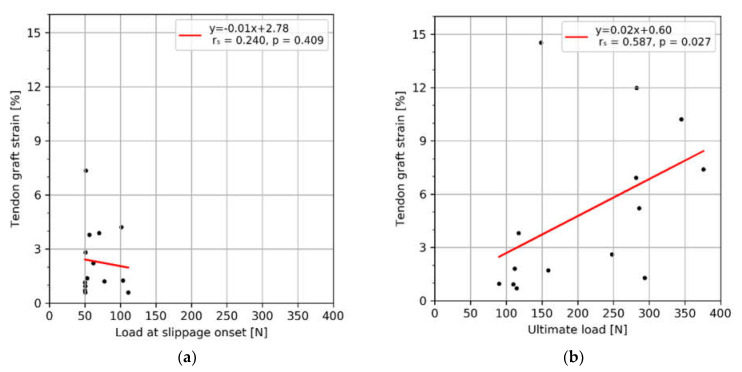
Correlation plots between the load and the tendon graft strain assessed at slippage onset (**a**) and ultimate load (**b**).

No significant correlation could be observed between the strain and the load at slippage onset (Figure 5a). The strain correlated positively with the ultimate load (Figure 5b).

## 4. Discussion

Fixation systems for ACL reconstructions have been extensively studied. Nevertheless, still show mostly inadequate mechanical properties affecting the restoration of physiological knee kinematics, graft motion, and integration. This study aimed to provide deeper insight into the experimental assessment of fixation stability in ACL reconstruction. By means of an optical motion tracking system, it was possible to directly measure graft strain, the displacement of the bone-specimen-embedding complex, as well as the graft displacement in different sites. This allowed the isolated assessment of actual graft slippage, independent of graft deformation. The independent measurement of tendon displacement allowed us to quantify the difference between the fixation stability measures defined by graft slippage i.e. the target output measure, and the machine actuator displacement, that is typically used as a standard method to calculate the stiffness of ACL fixation devices or methods in the literature.

The two main findings of this study are (i) the significant difference between displacements measured at the proximal, as well as the distal graft end and the machine actuator, and (ii) the significant correlations between displacmenets measured at different sites. Commonly, stiffness is calculated from a linear portion of the load-displacement curve as the ratio of the applied load and the corresponding deformation. In the literature, the displacement of a fixation in ACL reconstruction has usually been represented by the displacement of the load frame, i.e., machine actuator displacement (e.g., [12,13,26,27]). However, this approach does not result in a system stiffness assigned to fixation, but rather to a series of components, including the setup, embedding, bone specimen, tendon graft, and to the implant fixation itself. In the present study, the displacement of the distal tendon graft end remained close to zero until a certain load threshold was reached. Calculating the stiffness using machine actuator displacement data only would not provide specific information on the isolated structural characteristics of the implant fixation. More importantly, as the machine displacement includes the deformation of the tendon, using it to calculate stiffness would result in a significant underestimation of the fixation stiffness. However, the displacements measured at the different locations were found to correlate with each other. Hence, calculating stiffness using machine displacement would result in a precise, but inaccurate, prediction, allowing only for a qualitative comparison between the different fixation methods. To et al. [28] calculated the stiffness of different ACL fixations by modeling the tendon graft and fixation method as a series of springs. They reported the element stiffness of the graft to be 4- to 40-times higher than the element stiffness of femoral fixation methods. Stiffness is a crucial parameter for the restoration of physiological loads in the joint, as well as the stress and strain distributions of the articular cartilage [3]. Furthermore, finite element studies suggest that inadequate graft stiffness has an effect on graft tunnel enlargement and graft wear. Increasing the stiffness of the graft was found to increase stress at the contact zone between the graft and the sharp edges of the bone tunnel entrances [29]. Stiffness was not calculated in the present study because the load was applied cyclically until failure. Nevertheless, the ratio of the machine actuator versus tendon displacements at ultimate load was 3- to 4-fold that would proportionally reflect the stiffness. We assume this ratio to be greater for specimens from younger donors, as reported by To et al. (mean age: 64 years, ranging from 31 to 67 years).

In the present study, a high-resolution motion tracking system was used, which might not be available in other biomechanical laboratories. Alternatively, it would be feasible to track marks placed at the proximal tunnel end using a video camera, as performed by Mickelson et al. [21], and draw qualitative conclusions on graft slippage and stiffness. Graft motion at the proximal tunnel end is statistically significantly different from the distal tunnel end, but not at a clinically relevant level. Magen et al. [30] measured ACL graft slippage by computing the difference between the graft length after each complete loading cycle (at 10 N of preload) and its original length (at 10 N of preload). The residual displacement measured the combined effects of tendon graft stretch and fixation slippage, assuming the graft stretch was constant at a specified load. This effect was not observed in the present study. Residual displacement increased with higher loads due to the tendon’s viscoelastic behavior.

Besides stiffness, measuring the ultimate load, or the load at which the catastrophic failure occurs, has been established as the standardized method to quantify the primary stability of the ACL fixation systems. The ultimate load represents a clearly identifiable and easily reproducible variable that can be used to compare the strength of different fixation methods. However, the question has to be raised as to whether it is clinically relevant, i.e., if the fixation would be considered as failed when reaching the ultimate load or before, when slippage would cause a significant degree of laxity and instability of the knee joint. In the present study, the slippage at ultimate loads was 3.4 mm. While this is rather low, it is important to consider that, in some specimens, slippage reached clinically significant values (ranging up to 7.2 mm). Clinically, an additional concurrent slippage at the femoral side may be expected, leading to a higher degree of joint laxity. Therefore, the ultimate load alone may not be a sufficient clinically relevant indicator for fixation strength. On the basis of clinical investigations, biomechanical studies (e.g., [15,31]) have defined clinical failure at a 3-mm threshold of machine displacement. The data in the present study show, however, that this corresponds to less than one millimeter of actual graft slippage. Thus, a large portion of the reported displacement results from tendon elongation, rather than graft laxity.

While the tendon graft strain remained relatively low (3% at ultimate load), it showed high deviations, reached maxima of up to 15%, and it did not correlate with the applied tensile load. This finding indicates that tendon graft strain cannot be assumed as a constant variable across all specimens. Graft strain has a specimen-specific effect on the measured deformation and, furthermore, on the system stiffness. When targeting the assessment of fixation stability, the variation caused by graft compliance has to be regarded as a confounding factor.

In our study, the absolute displacement of the markers placed at the proximal end of the tibia in the direction of the applied load was a result of the possible relative displacement of the PMMA embedding in the load frame, the relative displacement of the bone in the embedding, or the elastic deformation of both components. However, all these displacements remained in the submillimeter range and were, compared to the displacement of the tendon and the machine actuator, negligibly small.

This study has limitations inherent to all biomechanical cadaveric investigations. In addition, it is limited by the high donor age, which does not fully reflect the typical patient cohort undergoing an ACL reconstruction. Furthermore, the applied unidirectional cyclic loading with increasing amplitude [17,18,19,20] does not necessarily replicate in vivo loading, with the latter remaining unknown. The simulation of accurate *in vivo* conditions in biomechanical studies is difficult and deviates from simplified models with simulated pathologies. The presented data are based on experiments with two different fixation screws, while there is a wide range of fixation devices and methods available. Other systems might show different slippage onset resulting in a different effect magnitude. The authors have chosen to perform the investigation using fixation screws because interference screw fixation is a frequently applied technique [2]. The authors suggest including commonly used devices in future research. Finally, the small sample size resulted in a large deviation of data, however with strong statistical significance between the displacements at different sites.

## 5. Conclusions

In conclusion, the experimental assessment of the stability following implant fixation methods in ACL reconstruction relies on the accurate measurement of the relative displacements and deformations of the tendon graft. The use of machine actuator displacement overestimates the true slippage of the graft because of the compliances of various components. However, it allows a qualitative comparison between different fixation methods and devices. If feasible, displacement should be assessed at the distal graft end to exclude artifactual graft elongation. The ultimate load can be used as a standardized variable for the comparison between different fixation methods. However, the ultimate load alone is not a sufficient measure to qualitatively describe fixation stability.

## Figures and Tables

**Table 1:** Results for all specimens for various displacement and graft strains, assessed at the slippage onset or ultimate load.

	Machine Displacement [mm]		Proximal Graft End Displacement [mm]		Distal Graft End Displacement [mm]		Abs. Vertical Bone Displacement [mm]		Graft Strain [%]	
Specimen ID	At Slippage Onset	At Ultimate Load	At Slippage Onset	At Ultimate Load	At Slippage Onset	At Ultimate Load	At Slippage Onset	At Ultimate Load	At Slippage Onset	At Ultimate Load
17709	2.5	6.7	1.4	2.8	0.1	0.7	0.0	0.0	1.2	1.7
17724	0.1	5.6	0.4	2.1	0.1	1.3	0.0	0.0	0.7	1.0
17725	0.1	13.4	0.4	6.2	0.1	6.1	0.1	1.0	0.6	2.6
17726	0.1	16.8	0.3	2.6	0.1	2.9	0.0	0.4	1.1	0.7
17733	2.0	9.5	1.4	4.1	0.1	2.4	0.0	0.2	0.6	1.3
17767	0.6	7.9	1.0	4.0	0.1	3.1	0.3	0.8	1.4	1.8
17768	2.0	23.4	1.6	8.4	0.1	7.2	0.2	1.2	4.2	10.2
17777	0.1	9.8	0.5	4.3	0.1	3.0	0.2	1.6	2.8	6.9
17791	0.2	21.6	0.4	6.3	0.1	5.0	0.0	0.1	0.9	0.9
17796	1.1	7.6	0.5	1.4	0.1	1.4	0.1	0.2	7.3	14.5
17798	1.6	16.0	1.0	6.9	0.1	4.9	0.2	0.9	1.3	5.2
17874	1.1	11.9	1.3	3.9	0.1	2.5	0.0	0.0	3.8	3.8
17875	0.9	11.4	0.6	2.9	0.1	3.9	0.1	0.2	2.2	12.0
17876	1.4	9.4	0.9	3.9	0.1	3.0	0.3	2.1	3.9	7.4
Mean ± SD (Min–MAX)	1.0 ± 0.8 (0.1–2.5)	12.2 ± 5.3 (5.6–23.4)	0.8 ± 0.4 (0.3–1.6)	4.3 ± 1.9 (1.4–8.4)	0.1 ± 0.0 (0.1–0.1)	3.4 ± 1.8 (0.7–7.2)	0.1 ± 0.1 (0.0–0.3)	0.6 ± 0.6 (0.0–2.1)	2.3 ± 1.9 (0.6–7.3)	5.0 ± 4.4 (0.7–14.5)

## Data Availability

The data presented in this study are available on request from the corresponding author. The data are not publicly available due to national privacy regulations.
